# Therapeutic Versus Preventative Use of *Ginkgo biloba* Extract (EGb 761) against Indomethacin-Induced Gastric Ulcer in Mice

**DOI:** 10.3390/molecules27175598

**Published:** 2022-08-31

**Authors:** Ahmed M. Abd-Eldayem, Sulaiman Mohammed Alnasser, Hanan H. Abd-Elhafeez, Soha A. Soliman, Rania A. Abdel-Emam

**Affiliations:** 1 Department of Medical Pharmacology, Faculty of Medicine, Assiut University, Assiut 71526, Egypt; 2 Department of Pharmacology and Toxicology, Unaizah College of Pharmacy, Qassim University, Buraidah 51452, Saudi Arabia; 3 Department of Cell and tissue, Faculty of Veterinary Medicine, Assiut University, Assiut 71526, Egypt; 4 Department of Histology, Faculty of Veterinary Medicine, South Valley University, Qena 83523, Egypt

**Keywords:** therapeutic, preventative, EGb 761, indomethacin, ulcer, mice

## Abstract

The main bioactive constituents in the standardized *Ginkgo biloba* leaf extract (EGb 761) are the terpene lactones and flavonoid glycosides. EGb 761’s antioxidant and anti-inflammatory properties have previously been demonstrated. Indomethacin-induced gastric ulcers have a multifactorial etiology and represent a major restriction to its therapeutic utility. The underlying ulcerogenic process involves oxidative and inflammatory biomolecular insults. This study was performed to explore the curative and preventative benefits of EGb 761 in experimentally-induced ulcers. To develop gastric ulcers in mice, indomethacin (40 mg/kg) was administered orally. EGb 761 (200 mg/kg) was given by gavage for 7 days before (preventative) and after (therapeutic) indomethacin administration. The histological alterations and macroscopic mucosal lesions were assessed. In gastric tissue homogenates, malondialdehyde (MDA), reduced glutathione (GSH), nitric oxide (NO), and inflammatory cytokines were measured. The expressions of cyclooxygenase-2 (COX-2), cytokines, and proliferating cell nuclear antigen (PCNA) in the stomach mucosa were also investigated. The ulcer index, histological alterations, gastric oxidants, and inflammatory biomarkers were all significantly increased by indomethacin. In stomach specimens, it increased COX-2 and PCNA expression. EGb 761 treatments, both prophylactic and therapeutic, resulted in significant reductions in ulcer lesions, nitrosative and oxidative damage, and inflammatory markers, along with the lowering of COX-2 and PCNA expressions. Furthermore, in the fight against stomach ulcers, EGb 761 treatment was found to be more efficient than prevention.

## 1. Introduction

Analgesic anti-inflammatory drugs (non-steroidal anti-inflammatory drugs, NSAIDs) are frequently used to mitigate pain, fever, and inflammation. They act by preventing the enzyme cyclooxygenase isoforms (COX) [1,2,3]. The physiologically expressed constitutive enzyme is COX-1, whereas COX-2 is an inducible enzyme that is anticipated within the inflammatory environment. Essentially, COX-1 retains the integrity of the gastric mucosa [4]. It produces cytoprotective prostaglandins that increase bicarbonate and mucus production, diminish gastric acid, and preserve proper mucosal blood flow [5]. It is widely accepted that NSAIDs raise the risk of gastric ulcers and gastric mucosal damage [6].

One of the non-selective NSAIDs is indomethacin, which has great efficacy allowing its usefulness in the treatment of tendinitis, osteoarthritis, and various inflammatory illnesses [7]. However, disruption of gastric mucosal integrity after its frequent use is the main concern of gastric ulcer development [8]. In comparison to other NSAIDs, indomethacin has a high ulcerogenic potential, encouraging its use for the induction of ulcers in experimental animals [9]. Studies show that the majority of people consistently take non-steroidal drugs, and a sizable proportion of them experience gastrointestinal side effects. [10].

As a prevalent upper gastrointestinal disorder, gastric ulcers have an elongated course, intricate therapy, and a high recurrence rate. They are characterized by deleterious outcomes such as perforation, bleeding, or even malignancy [11,12,13,14,15]. The etiology is assumed to be an imbalance between destructive and protective factors within the stomach [16]. The pathogenesis of indomethacin-induced gastropathy is underpinned by the excessive production of inflammatory cytokines and reactive oxygen species (ROS) [17,18]. Another major component of gastric ulceration is mucosal inflammation including nitric oxide (NO), inducible nitric oxide synthase (iNOS), COX-2, and 5-lipoxygenase (5-LOX) [19]. The gastric COX-2 gene was revealed to be significantly up-regulated in the indomethacin-treated mice [20,21,22]. Additionally, indomethacin-induced intestinal lesions [23,24] and ulcerated stomach and duodenal mucosa [25] were accompanied by up-regulated iNOS and COX-2 mRNA expression. On the other hand, some research, including various models of experimentally-induced ulcers, demonstrated the involvement of COX-2 in promoting the ulcer healing process [26,27,28]. 

Ulcer therapy can be difficult due to the multifaceted nature of the disease. Gastric ulcers have been treated with H2 receptor blockers, antibiotics, proton-pump inhibitors, and antacids. Gynecomastia, vitamin B12 deficiency, hypergastrinemia, hypoacidity, osteoporotic fracture, depression, and constipation have been documented as adverse effects [29]. As a result, safe, natural, and multitarget agents need to be developed [30,31,32,33]. In recent investigations, various natural compounds have been found as possible safe alternatives with low negative impacts as well as antioxidant and anti-inflammatory effects, which are beneficial against stomach ulcers [34,35,36].

*Ginkgo biloba L.* (Mantissa Plantarum Altera, 1771, Ginkgoceae) is a member of the Ginkgoceae botanical family, which includes *Salisburia adiantifolia, Salisburia macrophylla,* and *Pterophylla salisburiensis.* The *G. biloba* tree has thrived for over 150 million years in forests, earning it the name “living fossil”. [37,38]. 

The *G. biloba* tree contains sugars, amino acids, organic acids, polysaccharides, sterols and inositols. The active constituents have various chemical structures, including flavonoids, which comprise 26% of the mixture, terpenoids, which constitute 7%, and small amounts of organic acids. Flavonoids, often described as phenylbenzopyrones or phenylchromones, are a class of low molecular weight compounds that are found in a wide variety of plants. Brain illnesses, peripheral blood flow disorders, neurosensory syndromes, and cerebral insufficiency have all been treated with a well-defined and standardised mixture known as EGb 761 [39,40,41].

EGb 761, created by Dr. Wilmar Schwab Pharmaceuticals, has been used in Europe since the early 1990s. Nature’s Way in the United States distributes and markets a Ginkgo leaf standardized extract under the name Gingold Nature’s Way. EGb 761 is developed by Beaufour-Ipsen under the name Tanakan, Ipsen Pharma as Rökan, and Dr. Willmar Schwabe Pharmaceuticals as Tebonin. [38,42,43]. Flavonoids present in the Ginkgo leaf extract are flavonols, flavones, tannins, biflavones, and associated glycosides of quercetin and kaempferol attached to 3-rhamnosides, 3-rutinosides, or p-coumaric esters. The active components in the standardized *G. biloba* leaf extract (EGb 761) have antioxidant and anti-inflammatory properties [38,44]. 

It was discovered to be advantageous to use EGb 761 as a preventative agent against gastrointestinal damage caused by irradiation [45]. It has been demonstrated that EGb 761 protects against experimentally-induced stomach ulcers [46,47,48]. Pre-treatment with EGb 761 before ischemia-reperfusion was discovered to lower myeloperoxidase and malondialdehyde levels and diminish intestinal mucosal damage [49,50]. *G. biloba* extract inhibits stress-induced ulcer in rats [51]. Additionally, the extract showed gastric mucosal protective effects, reducing both the number and severity of gastric mucosal lesions induced by indomethacin in a dose-dependent manner [52]. In another study, administration of *G. biloba* ameliorated gastric lesions, with a significant decrease in ulcer score, MPO, and IL-1β and a significant rise in GSH, mucus content, and gastric pH [53].

In addition to determining how well EGb 761 works to prevent and treat indomethacin-induced stomach ulcers in mice, this study sought to clarify the impact of oxidative, nitrosative, and inflammatory biomolecules, as well as COX-2 and PCNA, in the development and progression of ulcers. Additionally, in order to determine whether EGb 761 should be used as a therapy or a preventative measure, we compared its potential therapeutic role with its preventive impact against gastric ulcers.

## 2. Results

### 2.1. Gastric Mucosal Lesions

**Figure 1:**
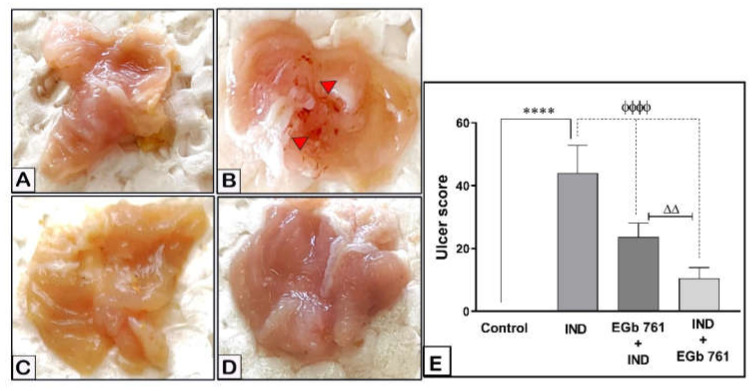
The effects of IND and EGb 761 on the gastric mucosa (macroscopic pictures and damage score). Results are represented as mean ± SEM (n = 6). (**A**) Control, (**B**) IND-induced ulcer, (**C**) EGb 761 pre-treatment, (**D**) EGb 761 treatment, (**E**) ulcer index scores. **** (*p* < 0.0001) significantly different from control. ^ϕϕϕϕ^ (*p* < 0.0001) significantly different from the ulcer group. ^ΔΔ^ (*p* < 0.01) significantly different from EGb 761+IND (Prophylactic EGb 761) group. IND: Indomethacin; EGb 761: Standardized G. biloba extract.

The gastric mucosa of mice, simply given the vehicle, revealed no visible damage. Comparative to the control group, the indomethacin-treated animals developed hemorrhagic ulcers, which showed significant macroscopic damage (Figure 1A,B). EGb 761 pre-treatment (Figure 1C) or treatment (Figure 1D) significantly reduced the resultant hemorrhagic damage of the mucosa. Damage score analysis revealed that indomethacin (40 mg/kg, orally) caused acute mucosal lesions in the stomach of the mice (UI = 44 ± 3.624, *p* < 0.0001). Compared to the IND group, EGb 761 pre-treatment or treatment resulted in lower stomach mucosal damage scores (UI = 23.67 ± 1.80, *p* < 0.0001) and (UI = 10.50 ± 1.41, *p* < 0.0001), respectively (Figure 1E). The ulcer score, as well as the preventive index (PI = 46.14%) and healing index (HI = 76.14%) values, suggest that EGb 761 has shown a strong healing impact, which was greater than its preventive use (*p* < 0.001).

### 2.2. Lipid Peroxidation (MDA Level)

**Figure 2:**
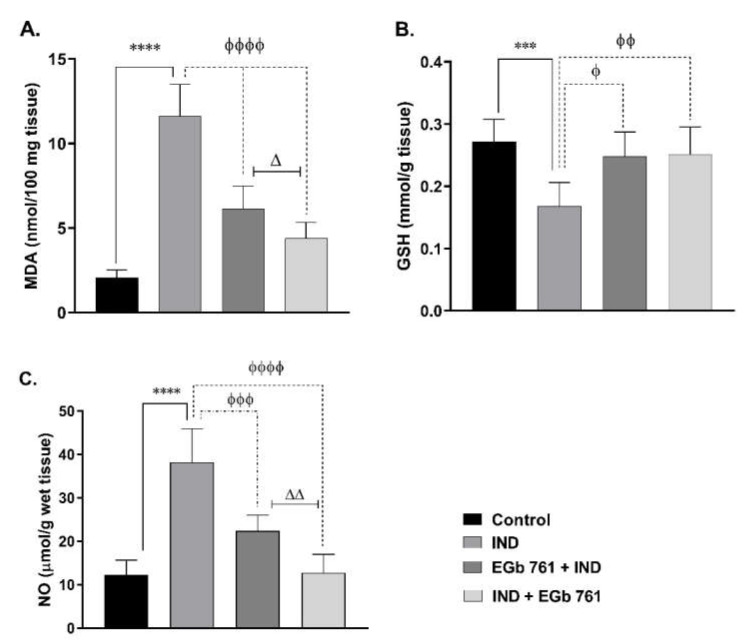
Changes were obtained by the administration of IND and EGb 761 on the gastric MDA tissue levels (**A**), GSH (**B**), and NO (**C**). Data are presented as mean ± SEM (n = 6). *** (*p* < 0.001) and **** (*p* < 0.0001) significantly different from control. ^ϕ^ (*p* < 0.05), ^ϕϕ^ (*p* < 0.01), ^ϕϕϕ^ (*p* < 0.001), and ^ϕϕϕϕ^ (*p* < 0.0001) significantly different from IND group. ^Δ^ (*p* < 0.05) and ^ΔΔ^ (*p* < 0.01) significantly different from EGb 761+ IND (Prophylactic EGb 761) group. IND: Indomethacin; EGb 761: Standardized *G. biloba* extract.

Oral administration of indomethacin produced a higher MDA level in the gastric tissue (11.63 ± 0.76 nmol/100 mg tissue, *p* < 0.0001) compared to the control mice, indicating a disturbed oxidant/antioxidant balance. In comparison to the IND group, EGb 761 pre-treatment substantially prevented an increase in MDA (6.13 ± 0.55 nmol/100 mg tissue, *p* < 0.0001), and EGb 761 treatment greatly lowered gastric the MDA level (4.40 ± 0.38 nmol/100 mg tissue, *p* < 0.0001). Treatment with EGb 761 resulted in a tremendous reduction in gastric MDA compared to EGb 761 pre-treatment (*p* < 0.05) (Figure 2A).

### 2.3. Gastric GSH Concentration

As illustrated in Figure 2B, the gastric tissue GSH of the indomethacin group, significantly, became 0.168 ± 0.015 mmol/g tissue (*p* < 0.001) in comparison to mice of the control group (0.272 ± 0.015 mmol/g tissue). EGb 761 pre-treatment and treatment were associated with an elevation in the gastric GSH levels close to its level in the stomachs of the control animals (0.248 ± 0.016 mmol/g tissue, *p* < 0.05; 0.252 ± 0.018 mmol/g tissue, *p* < 0.01, respectively) (Figure 2B).

### 2.4. NO Level

As indicated in Figure 2C, indomethacin induced a significant elevation in NO gastric contents (38.28 ± 3.14 mmol/g wet tissue, *p* ˂ 0.0001) compared to levels seen in gastric tissues from the vehicle-treated mice (12.33 ± 1.36 mmol/g wet tissue). Gastric NO levels were substantially reduced after pre-treatment and treatment with EGb 761 (22.42 ± 1.47 mmol/g wet tissue, *p* ˂ 0.001, and 12.78 ± 1.74 mmol/g wet tissue, *p* ˂ 0.0001, respectively (Figure 2C). The EGb 761 treatment group showed a significant reduction compared to the pre-treatment group (*p* ˂ 0.01).

### 2.5. Cytokine Levels

**Figure 3:**
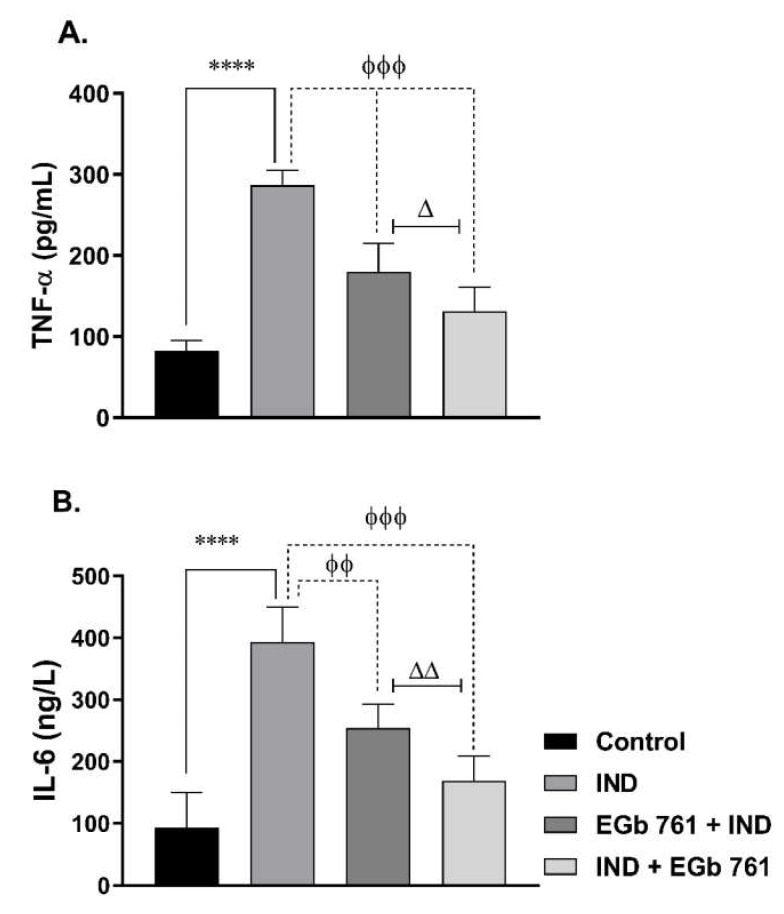
The effects of administration of indomethacin and EGb 761 on the gastric tissue levels of TNF-α (**A**) and IL-6 (**B**). Data are presented as mean ± SEM (n = 6). **** (*p* < 0.0001) significantly different from control. ^ϕϕ^ (*p* < 0.01) and ^ϕϕϕ^ (*p* < 0.001) significantly different from the IND group. ^Δ^ (*p* < 0.05) and ^ΔΔ^ (*p* < 0.01) significantly different from EGb 761 + IND (Prophylactic EGb 761) group. IND: Indomethacin; EGb 761: Standardized *G. biloba* extract.

Mice that were given indomethacin had significantly higher levels of TNF-α (286.7 ± 7.2 pg/mL) and higher levels of IL-6 (354.7 ± 21.29 ng/L) than the control mice (*p* < 0.0001) (Figure 3). When compared to the ulcer group, prophylactic treatment with EGb 761 attenuated the elevation of the gastric tissue cytokines; TNF-α (178.3 ± 9.72 pg/mL, *p* < 0.001) and IL-6 (230 ± 13.73 ng/L, *p* < 0.001). For mice that were treated with EGb 761 orally following indomethacin-induced ulcerations, the levels of TNF-α (173.7 ± 10.98 pg/mL, *p* < 0.001) and the levels of IL-6 (188.8 ± 21.71 ng/L, *p* < 0.01) were considerably lowered (Figure 3) as compared to the ulcer group. Additionally, Figure 3 shows that treatment with EGb 761 following indomethacin has a significant advantage over the pre-treatment group regarding TNF-α (*p* < 0.05) and IL-6 (*p* < 0.01) levels.

### 2.6. Histological Assessment

**Figure 4:**
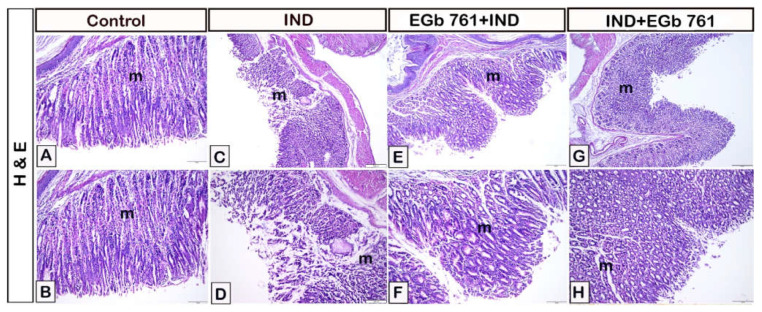
Gastric mucosal representative micrographs stained by hematoxylin and eosin. Effect of EGb 761 administration on the indomethacin-induced gastric ulcer. (**A**,**B**): Normal gastric mucosa in control mice. (**C**,**D**): Indomethacin-induced gastric ulceration (m) (IND). (**E**,**F**): EGb 761-pre-treated mice (Prophylactic EGb 761). (**G**,**H**): EGb 761 treatment after indomethacin (Therapeutic EGb 761). IND: Indomethacin; EGb 761: Standardized *G. biloba* extract.

Hematoxylin and eosin staining revealed normal gastric mucosa expression in the control animals (Figure 4A,B). Indomethacin-treated mice had stomach mucosal erosions and damage, as well as reduced gastric mucosal thickness, mucosal disruption, and inflammatory cell infiltration (Figure 4C,D). In stomach samples that had been pre-treated with EGb 761, the gastric mucosa was nearly intact (Figure 4E,F). Following ulcer induction, EGb 761 therapy resulted in near to normal gastric mucosa that was comparable to the control samples (Figure 4G,H). In Table 1, it was demonstrated that the administration of indomethacin resulted in significant gastric lesions, while administration of EGb 761 attenuated these induced lesions. In addition, the therapeutic administration of EGb 761 lowered the histological score more efficiently than prophylactic use.

### 2.7. Immunohistochemical Expression of COX2, IL-1β, TNF-α, IL-6, and PCNA

**Figure 5:**
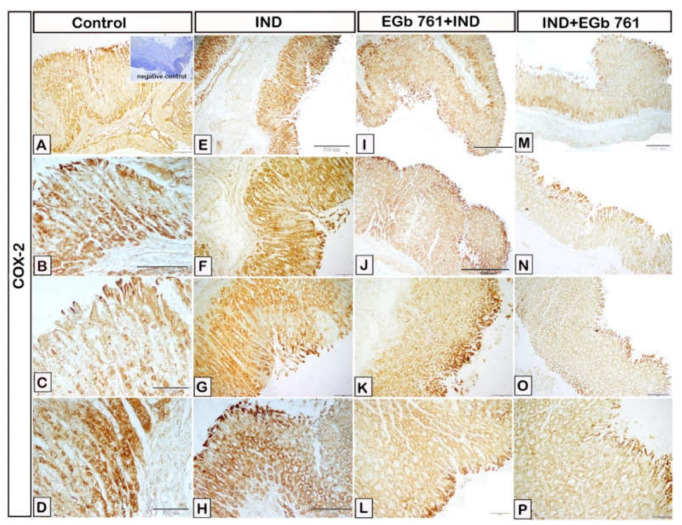
Photomicrograph immunohistochemistry of gastric mucosal expression of COX-2. (**A**–**D**): Control mice. (**E**–**H**): Indomethacin-induced ulcer (IND). (I-L): EGb 761 +IND (Prophylactic EGb 761). (**M**–**P**): IND+EGb 761 (Therapeutic EGb 761). Negative marker control is shown in the inset of Figure 5A. IND: Indomethacin; EGb 761: Standardized *G. biloba* extract; COX-2: Cyclooxygenase-2.

In the control samples, low expression of COX-2 was observed in the stomach epithelium (Figure 5A–D). The COX-2 expression was significantly higher within the cells of gastric mucosa in the indomethacin-induced ulcer samples as compared to the control mice (Figure 5E–H). A reduction in the expression of COX-2 was found in the gastric epithelium of stomach samples pre-treated with EGb 761 (Figure 5I–M). Figure 5N–Q shows a considerably low COX-2 immunoreactivity in the samples of gastric ulcers treated with EGb 761 when compared to the ulcer group.

**Figure 6:**
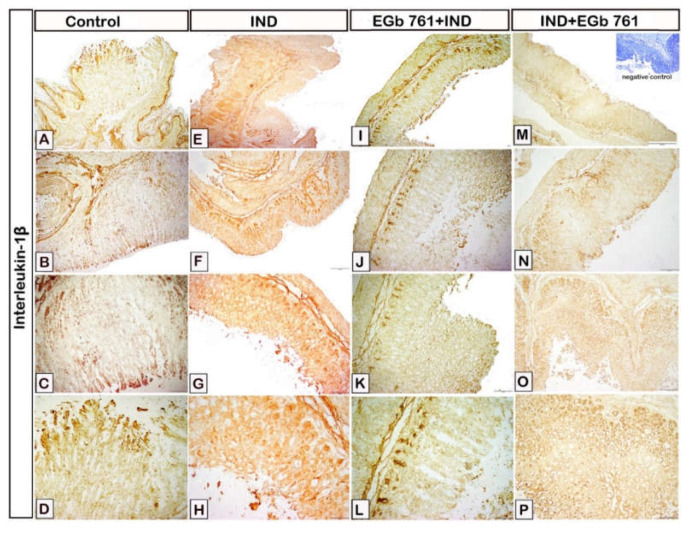
Photomicrograph immunohistochemistry of gastric mucosal samples shows the effects of indomethacin and EGb761 on gastric IL-1β expression. (**A**–**D**): Control mice. (**E**–**H**): Indomethacin-induced ulcer (IND). (**I**–**L**): EGb 761 +IND (Prophylactic EGb 761). (**M**–**P**): IND+EGb 761 (Therapeutic EGb 761). Negative marker control is shown in the inset of Figure 6M. IND: Indomethacin; EGb 761: Standardized *G. biloba* extract; IL-1β: Interleukin-1β.

Figure 6 displays the impact of indomethacin and EGb 761 on the immune reactivity of gastric IL-1β. Interleukin-1β expression was considerably greater in the indomethacin-treated group (Figure 6E–H) when compared to the control samples (Figure 6A–D). EGb 761 pre-treatment produced a dramatic decrease in IL-1β expression in the stomach epithelium (Figure 6I–L). EGb 761 treatment after ulcer development resulted in a substantial reduction in IL-1β expression when compared to the indomethacin group and EGb 761 pre-treated mice (Figure 6M–P).

**Figure 7:**
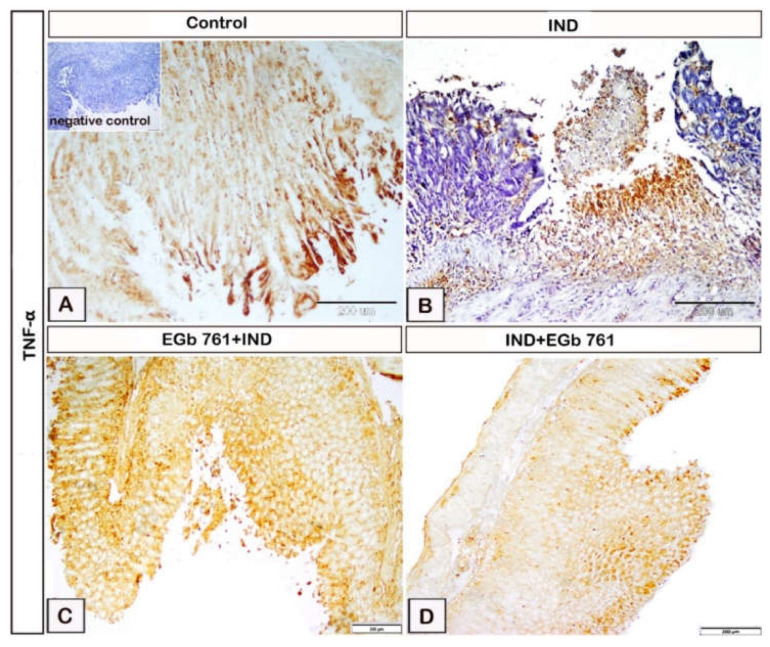
The changes in gastric TNF-α expression during indomethacin and EGb 761 administration were illustrated in photomicrograph immunohistochemistry of gastric mucosal samples. (**A**): Control mice. (**B**): Indomethacin-induced ulcer (IND). (**C**): EGb 761 +IND (Prophylactic EGb 761). (**D**): IND+EGb 761 (Therapeutic EGb 761). Negative marker control is shown in the inset of Figure 7A. IND: Indomethacin; EGb 761: Standardized *G. biloba* extract; TNF-α: Tumor necrosis factor-alpha.

The effects of indomethacin and EGb 761 regarding the expression of the gastric mucosal TNF-α are shown in Figure 7. In the control mice, weak TNF-α expression was observed (Figure 7A). Oral indomethacin administration resulted in a substantial increase in TNF-α expression in the gastric epithelium compared to the control mice (Figure 7B). EGb 761 pre-treatment was associated with a considerable decrease in TNF-α expression (Figure 7C). As compared to both the ulcer and the pre-treated group, mice treated with EGb 761 after indomethacin-induced ulceration exhibited a marked decrease in TNF-α expression (Figure 7D).

**Figure 8:**
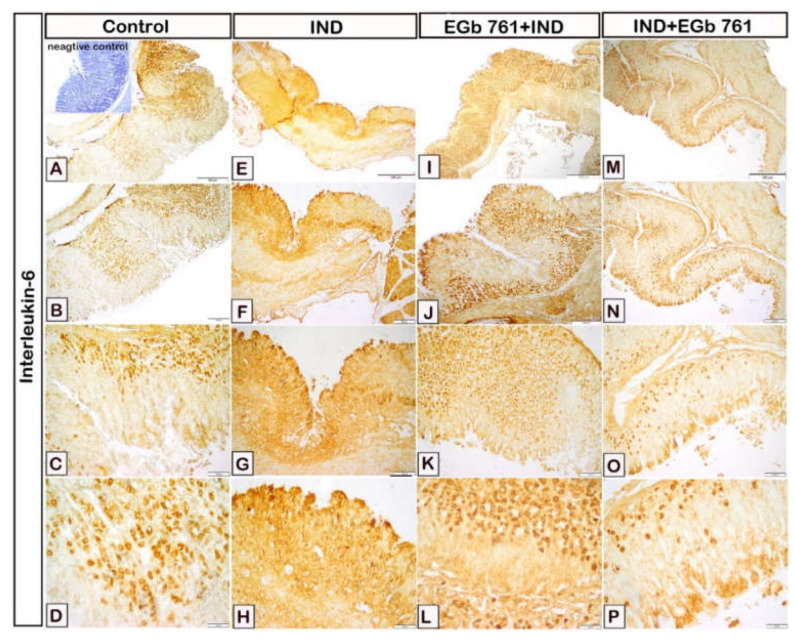
Immunohistochemical presentation of gastric tissue samples illustrating the effect of administration of indomethacin and EGb 761 on the expression levels of IL-6. (**A**–**D**): Control mice. (E-H): Indomethacin-induced ulcer (IND). (**I**–**L**): EGb 761 +IND (Prophylactic EGb 761). (**M**–**P**): IND+EGb 761 (Therapeutic EGb 761). Negative marker control is shown in the inset of Figure 8A. IND: Indomethacin; EGb 761: Standardized *G. biloba* extract; IL-6: Interleukin-6.

As displayed in Figure 8, the gastric mucosal expression of IL-6 was moderate in the stomach epithelium in the control samples (Figure 8A–D). Its expression was increased in the IND group with gastric ulceration as compared to the control mice (Figure 8E-H). In comparison to indomethacin-treated mice, IL-6 expression was significantly lowered with EGb 761 pre-treatment (Figure 8I–L). Additionally, when EGb 761 was administered following ulcer induction, IL-6 expression was likewise dramatically decreased (Figure 8M–P).

**Figure 9:**
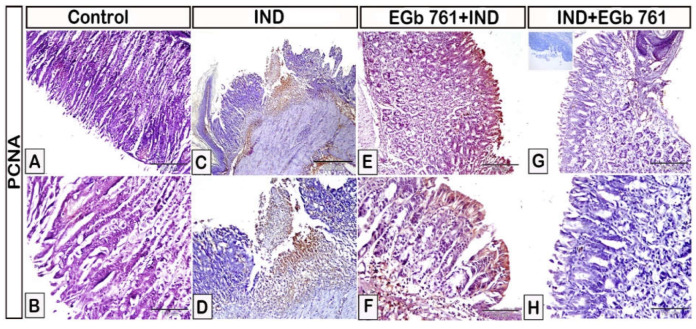
Photomicrograph immunohistochemistry of PCNA in the gastric mucosa. (**A**,**B**): Control mice. (**C**,**D**): Indomethacin-induced ulcer (IND). (**E**,**F**): EGb 761 +IND (Prophylactic EGb 761). (**G**,**H**): IND+EGb 761 (Therapeutic EGb 761). Negative marker control is shown in the inset of Figure 9G. IND: Indomethacin; EGb 761: Standardized *G. biloba* extract; PCNA: Proliferating Cell Nuclear Antigen.

The effects of indomethacin and EGb 761 on the gastric mucosal expression of PCNA is shown in Figure 9. The expression of gastric PCNA was significantly increased in mice with stomach ulcers, as shown in Figure 9C,D compared to the control animals that received the vehicle (Figure 9A,B). The expression of PCNA was lowered in stomachs of mice pre-treated with EGb 761 (Figure 9E,F). Administration of EGb 761 as a treatment following indomethacin was linked to a substantial reduction in gastric PCNA expression when compared to the IND and EGb 761 pre-treatment groups (Figure 9G,H).

Negative images of immunohistochemical investigations were provided for more illustration and confirmation of expressions of Cox-2 (Figure S1, Supplementary Materials), IL-1β (Figure S2, Supplementary Materials) IL-6 (Figure S3, Supplementary Materials), TNF-α (Figure S4, Supplementary Materials) and PCNA (Figure S5, Supplementary Materials).

### 2.8. Statistical Analysis of Area Percentage of Immunoreactivity of COX2, IL-1β, TNF-α, IL-6, and PCNA

**Figure 10:**
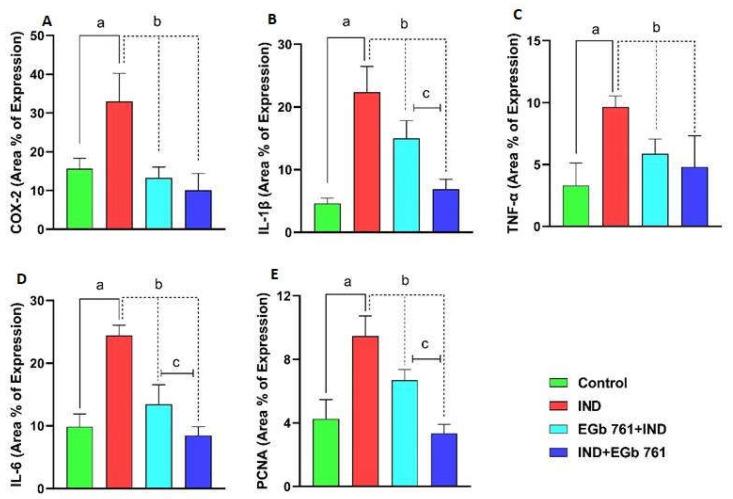
Area percentage values with statistical analysis regarding the expression of COX-2 (**A**), IL-1β (**B**), TNF-α (**C**), IL-6 (**D**), and PCNA (**E**) on immunohistochemical investigations of gastric mucosa in different groups. COX-2: Cyclooxygenase-2; IL-1β: Interleukin-1beta; TNF-α: Tumor necrosis factor-alpha; IL-6: Interleukin-6; PCNA: Proliferating Cell Nuclear Antigen. A high area percentage of expression values is noticed in the IND group (ulcer group). Prophylactic (EGb 761+IND) and therapeutic EGb 761 (IND+EGb 761) were associated with a dramatically low area percentage of expression. IL-1β, IL-6, and PCNA expressions were lower in therapeutic EGb 761 group when compared to prophylactic EGb 761. **a**: significant difference from control animals; **b**: significant difference from ulcer group; **c**: significant difference from prophylactic EGb 761 group.

Area percentage of immune expression of COX-2, IL-1β, TNF-α, IL-6, and PCNA shows that there is a major difference between groups (Figure 10A–E). The goal of this quantitative presentation of immunohistochemical analysis of the aforementioned markers is to demonstrate a meaningful difference between the ulcer group that received indomethacin and the control mice. In comparison to IND-treated mice, the immunoreactivity of the target markers was dramatically decreased when EGb 761 was administered before or after indomethacin. Furthermore, when the last two groups (pre-treatment and treatment) were examined, it was shown that EGb 761 therapy after ulcer formation was superior to pre-treatment use.

## 3. Discussion

In the prevention and treatment of numerous human ailments, including drug-induced stomach ulcers, herbal therapy has swiftly gained popularity. It has a greater priority in the selection of authorized chemical medications due to their high efficacy and safety. Their phytonutrient contents, along with their outstanding anti-inflammatory and antioxidant characteristics, have enabled them to play an important role in the treatment of toxicity disorders. *G. biloba* leaf extract (EGb 761) has been utilized as a traditional Chinese medicine for centuries to treat inflammatory diseases [54]. It has active ingredients with significant anti-inflammatory and antioxidant properties and a proven efficacy as a gastro-protective against harmful agents. In an earlier study, EGb 761 increased PGE2 levels, superoxide dismutase (SOD) activity, and reduced oxidative damage through cytoprotection and antioxidant actions to enhance the healing of the duodenal mucosa in duodenal ulcer rats [55]. EGb 761 consists of two main elements, flavonol glycosides (24%) (quercetin, kaempferol, isorhamnetin) and terpene lactones (6%) (3.1% are ginkgolides A, B, C, and J, and 2.9% is bilobalide) [56].

Intragastric administration of indomethacin to fasting mice resulted in macroscopically detected gastric ulcers that appeared as circular and linear hemorrhagic areas. The calculated ulcer score was significantly increased in comparison to the control mice. The biochemical measures further demonstrated a noticeable increase in lipid peroxidation, NO, and a decline in GSH levels in the gastric tissues of indomethacin-treated mice. Indomethacin-induced gastric tissue inflammation as evidenced by the significant elevation in tissue IL-1β, IL-6, and TNF-α levels was further supported by immunohistochemical analysis. Supporting evidence was documented in recent studies where indomethacin treatment significantly increased gastric MDA and decreased gastric antioxidant enzymes [46]. Indomethacin administration significantly elevated the gastric TNF-α and other inflammatory mediators with reduced gastric GSH levels [57]. Indomethacin up-regulated iNOS gene expression, NO level, and TNF-α levels in experimentally-induced gastrointestinal ulcerations [32,58].

The ulcerative mechanisms of indomethacin were attributed to the inhibition of COX-1. As a result, the protective mucus, PGE_2_, and bicarbonate are inhibited, increasing the belligerent factors, such as gastric acid and pepsin [59]. However, the role of oxidative stress and inflammation cannot be ruled out in the pathogenesis of indomethacin-mediated ulcer formation. Inflammation of the gastric mucosa and infiltration by neutrophils and macrophages represent the major source of ROS. These highly active free radicals induce gastric damage, increase MDA levels, and deplete enzymatic and non-enzymatic antioxidant defenses, such as GSH [60,61]. Nitrosative stress ensues when NO interacts with superoxide anions producing large amounts of peroxynitrite, a highly reactive nitrogen species, with major detrimental effects on gastric mucosal cells. Indomethacin induces the release of large amounts of NO by enhancing the expression of iNOS genes in the gastric mucosal cells. Increased expression of iNOS results in the stimulation of NF-κB, which augments the production of TNF-α and other pro-inflammatory cytokines [58]. 

In the current study, EGb 761 showed promising effectiveness as a prophylactic and therapeutic tool when used alone to alleviate indomethacin-induced gastric ulcers. Macroscopically, this was shown by the decline in the elevated ulcer index score. Moreover, the EGb 761 activity against the ulcers was much related to its ability to fight oxidative and inflammatory stresses. The antioxidant effect was manifested in our study by a decrease in the gastric levels of lipid peroxides (MDA) and NO, together with the significant preservation of GSH. Many studies investigating the gastroprotective effects of *G. biloba* extract have been conducted utilizing different methods for experimentally-induced gastric ulcers. These studies produced findings that met the outcomes obtained in this work [48,62,63,64]. By our results, Mahmoud [47] reported that EGb 761 induced a reduction in gastric NO and MDA in a model of ethanol-induced ulcers. El-Tanbouly et al. [46] documented that pre-treatment with EGb 761 reduced the gastric levels of MDA and serum C-reactive protein levels in indomethacin-treated rats.

Both flavonoid and ginkgolide components are involved in the antioxidant and free radical-scavenging capabilities of EGb 761, which reduce tissue ROS levels and stop membrane lipid peroxidation [65,66]. Kaempferol and Quercetin, two components of EGb 761, can lower iNOS mRNA expression in tissues and lower NO production in inflammatory and oxidative circumstances. 

The anti-inflammatory mechanism of EGb 761 was further displayed in our study by attenuating the IL-1β, TNF-α, and IL-6 gastric tissue contents. As an endogenous mediator, TNF-α has the potential to cause apoptosis and inflammation [67]. Supporting evidence was demonstrated in the previous study of Mahmoud [47], who recognized a substantial decrease in gastric TNF-α in animals with ethanol-induced gastric ulcerations. According to Li and his colleagues [68], the ginkgolides isolated from *G. biloba* leaves exhibited their anti-inflammatory activities through the inhibition of IL-6 and TNF-α production by suppressing the NF-κB gene expression. Moreover, the flavone glycosides as quercetin in EGb 761 showed anti-inflammatory merit through the prevention of IL-6 and TNF-α production [69]. Kaempferol dramatically reduced the extensive production of TNF-α and IL-6 in lipopolysaccharide-induced intestinal inflammation in rats [70]. 

Flavonoids activity to inhibit iNOS is greatly ascribed to inhibiting the induction of NF-κB [71]. According to Libermann and Baltimore [72], the promoter region of the IL-6 gene has a well-recognized binding site for NF-κB. Thus, the inhibition of NF-κB subsequently inhibits the gene transcription of IL-6, TNF-α, and other inflammatory cytokines [73]. Therefore, the inhibitory effect of EGb 761 on the expression of iNOS genes and NO production implies a dual antioxidant and anti-inflammatory action.

The free radical scavenging power of EGb 761 enables it to reduce the induced oxidative cellular damage and inflammation and augment the intracellular antioxidant defenses as GSH. Tang et al. [74] have shown that administration of EGb 761 was protective against myocardium ischemic/reperfusion injury by decreasing oxidative damage, suppressing inflammatory and NF-κB pathways. From the previous results, the mechanisms of ulcer induction by indomethacin and the anti-ulcer actions of EGb 761 could be explained.

Cyclooxygenase, also known as prostaglandin-endoperoxide synthase (PTGS), is an enzyme that produces prostanoids from arachidonic acid, such as thromboxane and prostaglandins such as prostacyclins [75]. The non-selective NSAIDs were found to have the same effect as selective COX-2 inhibitors in delaying experimental-induced gastric ulcer healing. The mRNA and protein expression of COX-2 are enhanced at the ulcer edges with enhanced proliferation of epithelial cells and growth factor expression, demonstrating a direct participation of COX-2 in the healing of ulcers [27,76]. COX-2 appears to increase the PGE2, which enhances tissue repair by inducing the production of growth factors in the gastric fibroblasts [77]. This might explain the enhanced expression of COX-2 in the gastric mucosal cells of indomethacin-treated mice.

The effect of indomethacin and EGb 761 on COX-2 expression during gastric ulceration was studied in this work. The expression of COX-2 was up-regulated in the indomethacin-treated mice, but down-regulated in the EGb 761-treated and pre-treated groups. This strong immunoreactivity to COX-2 in the ulcer model group was consistent with other studies [78,79]. EGb 761 therapy was found to suppress COX-2 expression in several studies, which were linked to its anti-inflammatory activity [80,81,82]. *G. biloba* extract’s expressional inhibitions of the protein and mRNA of both iNOS and COX-2 were related to NF-kB suppression [83].

Increased expression of PCNA in the stomach occurred in samples of indomethacin-induced gastric ulcerations, while its expression declined markedly in the EGb 761 pre-treated and treated groups. Gastric ulcers generated in the stomachs of rats by piroxicam administration demonstrated the degeneration of surface mucous cells as well as intense immunoreactivity to COX-2 and PCNA [79]. The PCNA expression was substantially higher in cases of gastritis and helicobacter pylori infection [14]. Its immunostaining reactivity was also dramatically increased in gastric ulcers induced by aspirin, which was considerably lower in the omeprazole-treated group [84]. Increased PCNA expression may be related to changes in cellular proliferation, which occurs as a compensatory reaction to mucosal injury. PCNA has a role in translesion synthesis, chromatin assembly, break-induced replication, and DNA replication-related processes [85].

Regarding the impact of EGb 761 on PCNA expression, Chao and Chu [55] found that *G. biloba* extract can significantly suppress PCNA expression in human hepatocellular carcinoma cells. The mild to moderate immunoreactivity to PCNA on EGb761 administration could be explained by EGb 761’s capacity to regenerate damaged tissues and normalise the proliferative process following re-epithelialization, differentiation, and effectiveness in healing gastric ulcers [79,86].

An exciting finding in the present work was that the therapeutic potential of EGb 761 in healing gastric ulceration induced by indomethacin was more successful than its prophylactic utility. This was evident from the observed values of the ulcer index, oxidative markers, and inflammatory cytokines in the damaged stomach mucosa of the treatment group in comparison to the pre-treated animals. The COX-2, IL-6, IL-1β, TNF-α, and PCNA expression was reduced in the EGb 761-treated mice more than in the pre-treated. This may indicate that EGb 761 is more effective to counteract the detrimental upper gastrointestinal effects of indomethacin as a therapeutic agent than it is prophylactically administered. 

## 4. Materials and Methods

### 4.1. Chemicals

In ordinary saline, indomethacin (Sigma-Aldrich, St. Louis, MO, USA) was dissolved. The Arab Company for Pharmaceuticals and Medicinal Plants, Egypt, provided the standardized *G. biloba* leaf extract (EGb 761) as a pure powder (Batch No.: 510421, Registration No.: 888/2011). It was dissolved to a final concentration of 40 mg/mL in 0.25% carbox-ymethylcellulose (CMC; Sigma-Aldrich, St. Louis, MO, USA) in saline. EGb 761 was heated and vortexed to assure its solubility before being stored at 4 C for 24 h prior to administration. According to Chassagne et al. [81], voucher specimens (GEO20494, GEO20496, and GEO20497) are kept at the Emory University Herbarium in Atlanta, Georgia, in the United States [87].

### 4.2. Experimental Animals and Study Design

The gastroprotective and therapeutic effects of EGb 761 were investigated using 24 male Swiss albino mice (25–30 g) obtained from the animal house of Assiut University. The mice were kept in a standard laboratory setting with an uninterrupted supply of food and water and a 12-hour light/dark cycle. To avoid coprophagia, the mice were maintained in cages with elevated flooring. The number of animals tested and the amount of ulcerogenic compounds used were the bare minimum needed to deliver reliable results. For all experimental methods, animals were handled in adherence to the Guidelines of the Care and Use of Laboratory Animals (1996; National Academy Press 2011, Washington, USA). Every procedure carried out during the study complied with the ethical guidelines set forth by the Faculty of Medicine at Assiut University for the humane care of animals, as well as all applicable legislation. The animal study protocol was approved by the ethical committee of the Faculty of Medicine, Assiut University (IRB: 17300796).

With minor modifications, the study was conducted in accordance with the methods of Athaydes et al. [88] and Pereira et al. [16]. The animals were starved for 24 hours before the induction of ulcers, although they were given full access to water. Animals were classified into four groups (n = 6). Oral indomethacin (IND) 40 mg/kg was administered to the ulcer group to induce gastric ulcers while the control mice were given the vehicle orally [88]. In the prophylactic group, animals were given EGb 761 (200 mg/kg, oral) [89,90] for 7 days prior to the administration of indomethacin (EGb 761+ IND). Mice that were given EGb 761 (200 mg/kg, oral) for 7 days following the indomethacin-induced ulcers represented the therapeutic group (IND + EGb 761).

Mice in the therapeutic group were euthanized at the end of the 7-day treatment period, whereas animals in the prophylactic group were anaesthetized and put to death with sodium thiopental (100 mg/kg, i.p.) 6 hours after receiving indomethacin. In order to inspect the ulcerative lesions, the stomachs were spread flat on a corkboard after being immediately opened along the larger curvature and gently cleansed with 0.9% saline solution.

### 4.3. Assessment of Gastric Mucosal Lesions 

To gauge the intensity of the gastric mucosal ulcers, the ulcer index (*UI*) was used. The damage score ranges from 0 for no lesions to 1 for petechiae, 2 for erosions less than 1 mm in length, 3 for erosions between 1 and 2 mm, 4 for erosions between 2 and 4 mm, and 5 for erosions longer than 4 mm. The ulcer index for each mouse under investigation was calculated by averaging the partial scores. The *UI* for each group was determined using the mean lesion score of all the animals in that group [91,92]. The following formulas were used to compute [93] the ulceration preventive index (*PI*) of the pre-treated group and the healing index (*HI*) of the treatment group as compared to the ulcer group.

(1) $$PI=\frac{\left(\mathrm{UI}\;\mathrm{ulcer}–\mathrm{UI}\;\mathrm{pre}-\mathrm{treated}\right)}{\mathrm{UI}\;\mathrm{ulcer}}\times 100$$

(2) $$HI=\frac{\left(UI\;ulcer–UI\;treated\right)}{UI\;ulcer}\times 100$$

### 4.4. Determination of Lipid Peroxidation (TBARS) 

The thiobarbituric acid reactive substance was measured using the described method [94,95]. By determining the amount of malondialdehyde (MDA) produced, and measuring it using a chromogen at 532 nm, the intensity of lipid peroxidation was detected spectrophotometrically.

### 4.5. Determination of Gastric Reduced Glutathione Concentration 

Spectrophotometric analysis was used to gauge the glutathione levels in the gastric tissue. Reducing 5,5’-dithiobis (2-nitrobenzoic acid) (DTNB) with glutathione results in a yellow molecule (GSH). The GSH concentration is directly proportional to the reduced chromogen’s absorbance, which can be measured at 405 nm. The GSH concentration was calculated using the following equation:

(3) $$\mathrm{GSH}\left(\mathrm{mmol}/g.\mathrm{tissue}\right)=\frac{A\;\mathrm{sample}\times 2.22}{g.\mathrm{tissue}}$$

### 4.6. Nitric Oxide (NO) Measurement

The amount of nitrite in the gastric tissue was calculated by converting nitrate to nitrite using vanadium trichloride (VCl3) and then adding Griess reagent [96]. Spectrophotometric analysis was used to determine the absorbance at 540 nm after 30 minutes of incubation at 37 °C. By comparing results to sodium nitrite standards that were simultaneously measured and shown on a standard curve, the concentration of nitrite in each sample was identified and expressed as nmol/g tissue.

### 4.7. Determination of Tumor Necrosis Factor-alpha (TNF-α) and Interleukin-6 (IL-6)

The gastric tissue homogenate was used for the measurement of the level of inflammatory markers (IL-6 and TNF-α). This was performed utilizing commercial ELISA kits. To determine the TNF level, the AssayMax mouse Tumor Necrosis Factor-ELISA Kit of murine monoclonal antibody was used (AssayPro, St. Charles, MO, United States, Catalog Number: ERT2010-1). A commercial IL-6 ELISA kit was used following the manufacturer’s instructions for the estimation of the gastric level of IL-6 (BT Lab, Nanhu Dist, Jiaxing, Zhejiang, China, Catalog number: E0049Mo). The values are presented as the mean ± standard error. All kits implemented the sandwich technique of ELISA, based on the manufacturers’ instructions.

### 4.8. Histopathological and Immunohistochemical Evaluation

#### 4.8.1. Collection of Samples and Preparation for Paraffin Embedding

Three small stomach tissue specimens (approximately 1 cm^3^) were obtained from mice in each group: control, indomethacin, EGb 761 pre-treatment, and EGb 761 treatment group. The tissue specimens were fixed in the following fixative: 40 mL paraformaldehyde, 25% freshly made, 125 mL phosphate buffer (0.2 M, pH 7.4), 37.5 mL saturated picric acid, 0.5 mg calcium chloride, 1.25 mL glutaraldehyde 25%, and distilled water was added up to 250 mL. The samples were fixed using Wrobel–Moustafa fixative for 24 h [97,98,99]. Samples were cleaned to remove the fixative prior to processing. The fixed samples were thoroughly washed with 70% ethanol three times over the course of 24 hours. Following cleaning, samples were encased in paraffin wax. Using a Reichert Leica RM2125 Microtome, sections were cut between 5 and 7 m. Hematoxylin and eosin was used to stain paraffin representative sections for general histological study and scoring (H&E).

#### 4.8.2. The Immunohistochemical Procedure of COX-2, IL-1β, TNF-α, IL-6, and PCNA

We carried out two-step immunohistochemical staining using Dako EnVision+ Single Reagent (HRP. Mouse; Agilent Technologies, Inc., Santa Clara, CA, USA) [100]; a staining strategy should be used, according to Abd-Elhafeez et al. [101]. The 5-m-thick paraffin-embedded sections were dewaxed, rehydrated, and PBS-washed (pH 7.4, three times, for 5 min each time, Table 2). Following the suppression of endogenous peroxidase activity, sections were thoroughly washed in PBS before being heated in the microwave to retrieve antigens (Table 2) and allowing them to cool for 20 minutes at room temperature. The sections were exposed to Dako Protein Block (Agilent Technologies, Inc., Santa Clara, CA, USA) for 5 min. Primary antibodies were incubated on the slides (Table 2). Slides were incubated for 30 min with the secondary antibody, followed by a wash (Table 3). Following a PBS wash, the slides underwent a DAB and substrate-chromogen treatment from 5 to 10 min. The sections were dehydrated in ethanol and counterstained with Harris hematoxylin. To assess immunohistochemical staining, we used a Leitz Dialux 20 microscope and a Canon PowerShot A95 digital camera.

#### 4.8.3. Detection of Area Percentage of Immune Expression of COX-2, IL-1β, TNF-α, IL6, PCNA, and statistical analysis 

Image J was used to calculate the area percentage of immunohistochemistry pictures as the following: Using image J Fiji software, open each image one by one. Convert the image to an 8-bit image from the image column, then go to “type” and select “8-bit”, then go to “analyze column” and select the measurement, then check the area and area fraction and set “okay”, then go to “image” and select “adjust” then select “threshold”. From the pull-down menus, choose default, red, and dark backgrounds. Move the top slider until the entire foreground is red to threshold the image. Attempt to maintain the stained area as uniformly as possible. When finished, click “Apply”. For quantification of immunohistochemistry images using image J and how to remove background in image J, follow the provided link: https://www.google.com/search?q=quantification+of+immunohistochemistry+images+using+imagej+%7C+how+to+remove+background+in+imagej&rlz=1C1GCEA_enEG992EG992&oq=q&aqs=chrome.1.69i57j35i39j0i131i433i512j46i199i291i433i512j0i433i512l2j46i433i512j0i512j0i131i433i512j46i131i199i433i465i512.2237j0j15&sourceid=chrome&ie=UTF-8 (accessed on 15 October 2021).

#### 4.8.4. Color Segmentation by CMEIAS (The Negative Images in the Supplementary Figures)

Negative images were produced using CMEIAS Color Segmentation, a cost-free, enhanced computational technique. To do this, the subsequent actions were taken: In CMEIAS Color Segmentation, open the image and select “Process” from the drop-down menu. The drop-down choice will say “Negative photo”, so choose that [101]. All supplementary figures are demonstrated in supplementary files showing negative images of immunohistochemistry stains.

### 4.9. Statistics

All values were represented as the mean ± standard error. Statistical assessment was conducted using GraphPad Prism 7 software (One-way ANOVA with a post-hoc Tukey’s correction) and the SPSS program (version 17) (One-way ANOVA, followed by the Scheffe and Duncan test). To identify any significant differences between the pre-treatment and treatment groups, a T-test was applied. A *p*-value < 0.05 was deemed significant.

## 5. Conclusions

There is a pressing need for a secure treatment for stomach ulcers, particularly those caused by drugs, and there have been several attempts to achieve this. It would be optimal if these medications came from a natural source. In this work, we examined the function of EGb 761 in indomethacin-induced stomach ulcers. We were looking to see if there were any differences between using EGb 761 to prevent and treat ulcers. The results showed that EGb 761 could help prevent and treat stomach ulcers brought on by indomethacin. Its antioxidant and anti-inflammatory properties are linked with these advantageous effects. Histopathological and biochemical results support this conclusion. More so than in the pre-treated animals, the EGb 761-treated groups showed improved score, levels, and expressions of the ulcer index, MDA, GSH, NO, IL-6, TNF-α, IL-1β, COX-2, and PCNA. Our findings recommend that it is more advantageous to use EGb 761 as a therapeutic rather than a preventive therapy against indomethacin-induced stomach ulcers and mucosal lesions. 

## Figures and Tables

**Table 1:** Effect of EGb 761 on gastric tissue histomorphology of indomethacin-induced gastric ulcer.

Histopathological Lesions	Control	Indomethacin-Induced Gastric Ulceration	EGb 761 Pre-Treated Mice	EGb 761 Treatment after Indomethacin
**Necrosis of Gastric** **Mucosa**	00	04	02	01
**Mucosal Inflammatory** **Cells**	00	03	02	01
**Submucosal Oedema**	00	04	02	01
**Hemorrhage**	00	04	01	01
**Total Score**	**00**	**15**	**07**	**04**

**Table 2:** Components of the fixatives.

Fixative	Components	Amount
N a-Phosphate buffer (0.1 M, pH 7.4)	Solution A	
	Na2HPO4 2H2O	17.02 gm
	Distilled water	600 mL
	Solution B	
	NaH2PO4 H2	6 gm
	Distilled water	200 mL
	Using solution	
	Solution A	580 mL
	Solution B	219 mL
Citrate-buffer (pH 6.0)	Solution A	
	Citrate C6H8O7 H2O	21 g
	Distilled water	1 L
	Solution B	
	Sodium citrate Na3C6H5O7 2H2O	29.41 g
	Distilled water	1 L
	Using solution	
	Solution A	9 mL
	Solution B	41 mL
	Distilled water	Add 500 mL

**Table 3:** Identity, sources, and the working dilution of antibodies used in immunohistochemical studies.

Target	Primary Antibody Supplier	Origin (Catalog No)	Dilution	Incubation	Antigen Retrieval	Secondary Antibody-Incubation Time
COX-2	Abcam	Rabbit anti-mouse monoclonal [EPR12012] to COX2	1:100	Overnight	Boiling in citrate buffer (pH 6.0), 20 min Goat	Goat anti-Mouse IgG (H+L) Secondary Antibody Catalog # 31569 Dilution; 1:100 One hour at room temperature
IL- 1β	Bio-Rad	Rabbit anti-Mouse Interleukin-1 beta Clone: AAM13G Polyclonal	1:200	Overnight	Boiling in citrate buffer (pH 6.0), 20 min	
IL-6	Abcam	Mouse monoclonal Anti-IL-6 antibody [1.2-2B11-2G10] (ab9324)	1:100	45-min incubation at room temperature	Boiling in citrate buffer (pH 6.0), 20 min	
TNF-α	(Novus Biologicals, Littleton, CO, USA),	TNF-alpha Antibody [NBP1-19532]	1:300	Overnight	Boiling in citrate buffer (pH 6.0), 20 min	
PCNA	Anti PCNA Santa Cruz Biotechnology, Inc.	(PC10 sc-56 mouse anti-rat IgG2a monoclonal antibody	1:500	2 h at room temperature	Boiling in citrate buffer (pH 6.0), 20 min	

## Data Availability

The data presented in this study are available upon request from the corresponding author.

## Supplementary Materials

The following supporting information can be downloaded at: https://www.mdpi.com/article/10.3390/molecules27175598/s1, Figure S1: the negative image of COX-2 expression. Figure S2: the negative image of IL-1β expression. Figure S3: the negative image of IL-6 expression. Figure S4: the negative image of TNF-α expression. Figure S5: the negative image of PCNA expression.

## Sample Availability

Samples of the compounds are available from the authors.
