# Supplementary material for: Therapeutic Versus Preventative Use of Ginkgo biloba Extract (EGb 761) against Indomethacin-Induced Gastric Ulcer in Mice
Source: Molecules. 2022 Aug 31;27(17):5598. doi: 10.3390/molecules27175598 (PMC9458100; doi:10.3390/molecules27175598)
Supplement: Supplementary file 1 [file molecules-27-05598-s001.zip › METHODS X.pdf]

METHODS X

Assessment of gastric mucosal lesions

The ulcer index (UI) was used to measure the severity of gastric mucosal ulcers. The damage score is 0 for no lesions, 1 for petechiae, 2 for erosions less than 1 mm, 3 for erosions between 1 and 2 mm, 4 for erosions between 2 and 4 mm, and 5 for erosions greater than 4 mm in length. The ulcer index of each investigated mouse was calculated by adding the partial scores obtained. The mean lesion score of all mice in each group was used to calculate the UI for that group. The ulceration preventive index (PI) of the pre-treated group and the healing index (HI) of the treatment group *vs* the ulcer group were calculated using the following formulae.

$$PI = (UI_{ulcer} – UI_{pre\text{-}treated}) / UI_{ulcer} \times 100.$$

$$HI = (UI_{ulcer} – UI_{treated}) / UI_{ulcer} \times 100.$$

Determination of lipid peroxidation (TBARS)

The thiobarbituric acid reactive substance was measured using the described method. By determining the amount of malondialdehyde (MDA) produced and measuring it using a chromogen at 532 nm, the intensity of lipid peroxidation was detected spectrophotometrically. A 100 µL of stomach homogenate was combined with 200 µL sodium dodecyl sulfate 10% (SDS) and 250 µL of the color reagent (thiobarbituric acid 0.037 % + hydrochloric acid 0.25 M + trichloroacetic acid 15%) in microtubes. The mixture was heated in the dark for 15 minutes at 95°C before being cooled for 5 minutes. The microtubes were then centrifuged at 3500 rpm for 4 minutes at 4 °C, and the supernatant was deposited in a 96-well microplate and measured at 532 nm with a microplate reader.

Determination of gastric reduced glutathione concentration

Glutathione levels in gastric tissue were measured spectrophotometrically. The method produces a yellow compound by reducing 5,5' dithiobis (2-nitrobenzoic acid) (DTNB) with glutathione (GSH). The reduced chromogen's absorbance can be measured at 405 nm and is directly proportional to GSH content. A 0.5 ml of trichloroacetic acid was added to 0.5 ml of fresh tissue homogenate and allowed to stand for 5 minutes at room temperature before centrifugation at 3000 rpm for 15 minutes. A 0.5 mL supernatant was combined with 1.0 mL buffer and 0.1 mL DTNB in a mixture. After mixing, an appropriate volume was carefully transferred to a microplate and incubated for 10 minutes before measuring the absorbance (A) with a microplate reader at 405 nm. The following equation was used to determine the GSH concentration:

GSH (mmol / g. tissue) = A_{Sample} x 2.22/ g. tissue

Nitric oxide (NO) measurement

The gastric tissue nitrite level was measured by a procedure including the conversion of nitrate to nitrite by vanadium trichloride (VCl3) followed by the addition of Griess reagent. In brief, 0.5 ml of tissue homogenate was combined with Griess reagent and an equal volume of VCl3 (400 mg dissolved in 50 ml 1 M HCl). Spectrophotometrically, the absorbance was measured at 540 nm after 30 minutes of incubation at 37°C. By comparing values to sodium nitrite standards that were measured in parallel and displayed on a standard curve, the nitrite concentration in each sample was calculated. The NO concentration was represented as nmol/g tissue.

4.8.3. Detection of area percentage of immune expression of COX-2, IL-1β, TNF-α, IL6, PCNA, and statistical analysis

Image J was used to calculate the area percentage of immunohistochemistry pictures as the followings. Using image J Fiji software, open each image one by one. Convert image to 8-bit image from image column, then go to type and select 8 bit, then go to Analyze column and select-set measurement-then check area and area fraction and set okay, then go to Image- and select adjust-then select threshold. From the pull-down menus, choose default, red, and dark backgrounds. Move the top slider until the entire foreground is red to threshold the image. Attempt to maintain the stained area as uniform as possible. When you're finished, click "Apply." For quantification of immunohistochemistry images using image J and how to remove background in image J follow the following link:

https://www.google.com/search?q=quantification+of+immunohistochemistry+images+using+imagej+%7C+how+to+remove+background+in+imagej&rlz=1C1GCEA_enEG992EG992&oq=q&aqs=chrome.1.69i57j35i39j0i131i433i512j46i199i291i433i512j0i433i512l2j46i433i512j0i512j0i131i433i512j46i131i199i433i465i512.2237j0j15&sourceid=chrome&ie=UTF-8.

Table S1: Components of the fixatives

Fixative	Components		Amount
N a-Phosphate buffer (0.1 M, pH 7.4)	Solution A		
		Na2HPO4 2H2O	17.02 gm
		Distilled water	600 ml
	Solution B		
		NaH2PO4 H2	6 gm
		Distilled water	200 ml
	Using solution		
		Solution A	580 ml
		Solution B	219 ml
Citrate-buffer (pH 6.0)	Solution A		
		Citrate C6H8O7 H2O	21 g

	Distilled water	1 liter
Solution B		
	Sodium citrate Na3C6H5O7 2H2O	29.41 g
	Distilled water	1 liter
Using solution		
	Solution A	9 ml
	Solution B	41 ml
	Distilled water	Add 500 ml

TableS2: Identity, sources, and the working dilution of antibodies used in immunohistochemical studies

Target	Primary antibody supplier	Origin (catalog no)	Dilution	Incubation	Antigen retrieval	Secondary antibody-incubation time
COX-2	Abcam	Rabbit anti-mouse monoclonal [EPR12012] to COX2	1:100	Overnight	boiling in citrate buffer (pH 6.0), 20 min Goat	Goat anti-Mouse IgG (H+L) Secondary Antibody Catalog #
IL- 1β	Bio-Rad	Rabbit anti-Mouse Interleukin-1 beta Clone: AAM13G Polyclonal	1:200	Overnight	boiling in citrate buffer (pH 6.0), 20 min	31569<br><br>Dilution; 1:100 One hour at room temperature
IL-6	Abcam	Mouse monoclonal Anti-IL-6 antibody [1.2-2B11-2G10] (ab9324)	1:100	45-minute incubation at room temperature	boiling in citrate buffer (pH 6.0), 20 min	
TNF-α	(Novus Biologicals, Littleton, CO, USA),	TNF-alpha Antibody [NBP1-19532]	1:300	Overnight	boiling in citrate buffer (pH 6.0), 20 min	
PCNA	Anti PCNA Santa Cruz Biotechnology, Inc.	(PC10 sc-56 mouse anti-rat IgG2a monoclonal antibody,	1:500	2 hours at room temperature	boiling in citrate buffer (pH 6.0), 20 min	

4.8.4. Color Segmentation by CMEIAS (for the negative images of supplementary figures)

CMEIAS Color Segmentation, a free, improved computing method, was used to create negative images. The following procedures were taken to accomplish this: open the image in CMEIAS Color Segmentation, then "Process" from the drop-down menu. Select "Negative picture" from the drop-down menu [101]. All supplementary figures are demonstrated in supplementary files showing negative images of immunohistochemistry stains.
