# Supplementary material for: Therapeutic Versus Preventative Use of Ginkgo biloba Extract (EGb 761) against Indomethacin-Induced Gastric Ulcer in Mice
Source: Molecules. 2022 Aug 31;27(17):5598. doi: 10.3390/molecules27175598 (PMC9458100; doi:10.3390/molecules27175598)
Supplement: Supplementary file 1 [file molecules-27-05598-s001.zip › suppl file new.pdf]

Therapeutic versus preventative use of Ginkgo Biloba Extract (EGb 761) against indomethacin-induced gastric ulcer in mice.

Ahmed M. Abd-Eldayem[1], Sulaiman Mohammed Alnasser[2], Hanan H. Abd-Elhafeez[3], Soha A. Soliman[4], Rania A. Abdel-Emam[1].

[1] Department of Pharmacology, Faculty of Medicine, Assiut University, Assiut, 71526, Egypt.

[2]Department of pharmacology and toxicology, Unaizah College of Pharmacy, Qassim University, Qassim, Saudi Arabia.

[3]Department of Cell and Tissue, Faculty of Veterinary Medicine, Assiut University, Assiut, 71526, Egypt.

[4]Department of Histology, Faculty of Veterinary Medicine, South Valley University, Qena, 83523, Egypt.

Correspondence

Sulaiman Mohammed Alnasser: Department of pharmacology and toxicology, Unaizah College of Pharmacy, Qassim University, Qassim, Saudi Arabia. Postal Code: 51911, tel: 00966541841155, Email: sm.alnasser@qu.edu.sa

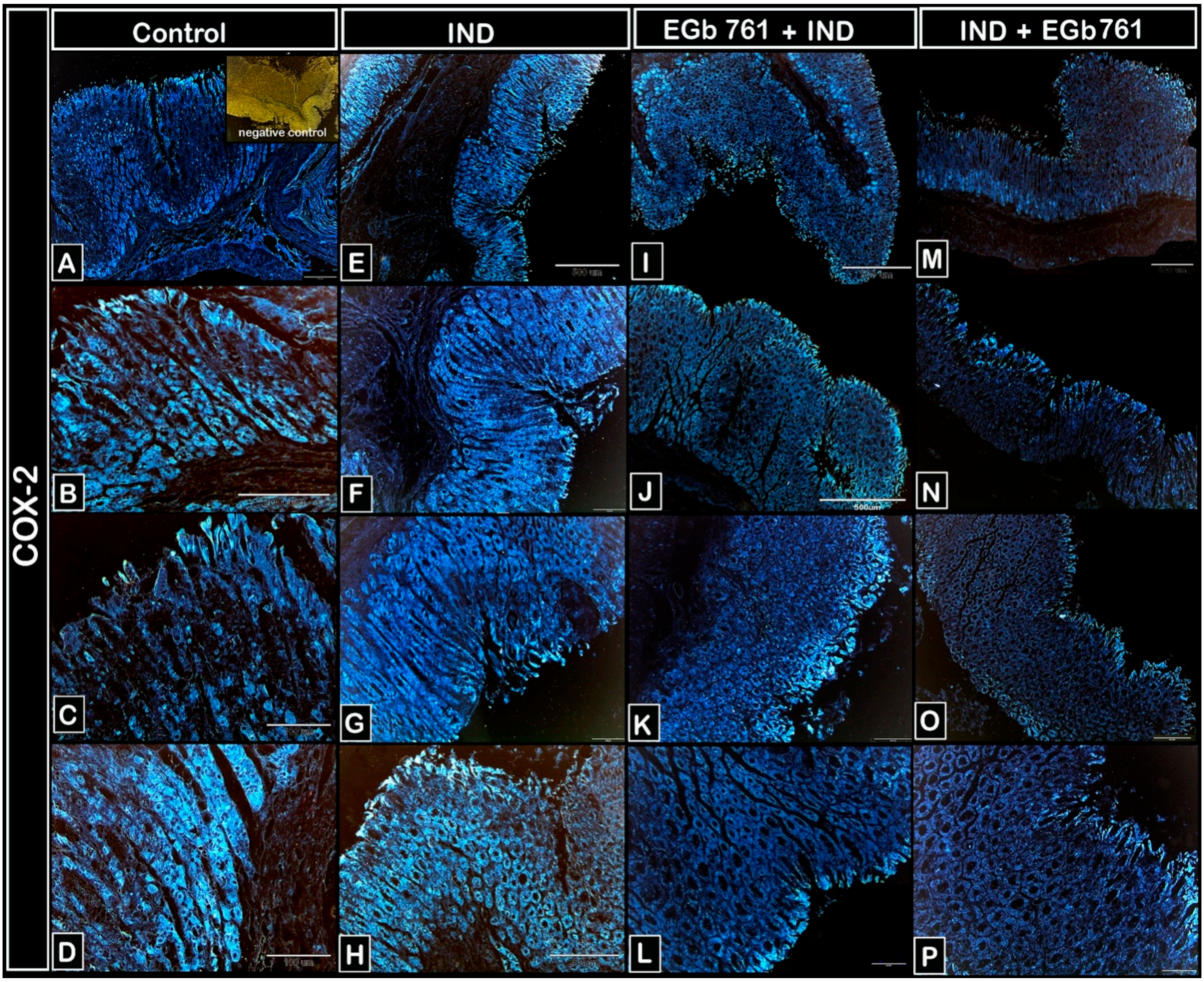

Figure S1. The negative image of the effect of indomethacin or EGb 761 on COX-2 expression in gastric mucosa. (A-D): Control mice, (E-H): Indomethacin-induced ulcer (IND), (I-L): EGb 761 pre-treatment (EGb 761+IND), (M-P): EGb 761 treatment after indomethacin (IND+EGb-761). Negative marker control is shown in the inset of figure A. IND: indomethacin, EGb 761: Standardized Gingko biloba extract, COX-2: Cyclooxygenase-2.

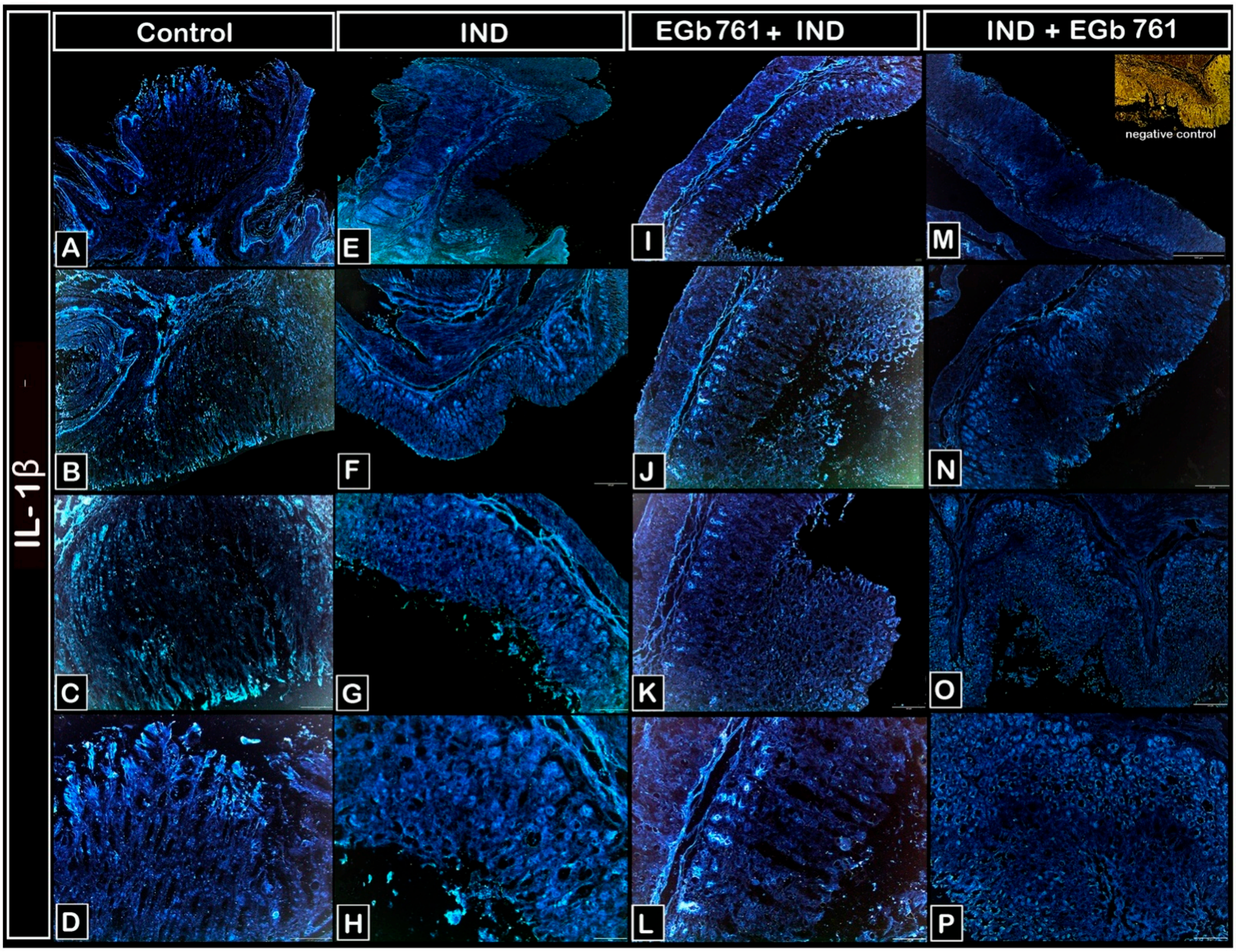

Figure S2. The negative image of the effect of indomethacin and EGb 761 on the expression of gastric IL-1β. (A-D): Control mice, (E-H): Indomethacin-induced ulcer (IND), (I-L): EGb 761 pre-treatment (EGb 761+IND), (M-P): EGb 761 treatment after indomethacin (IND+EGb 761). Negative marker control is shown in the inset of figure M. IND: Indomethacin, EGb 761: Standardized Gingko biloba extract, IL-1β: Interleukin-1β.

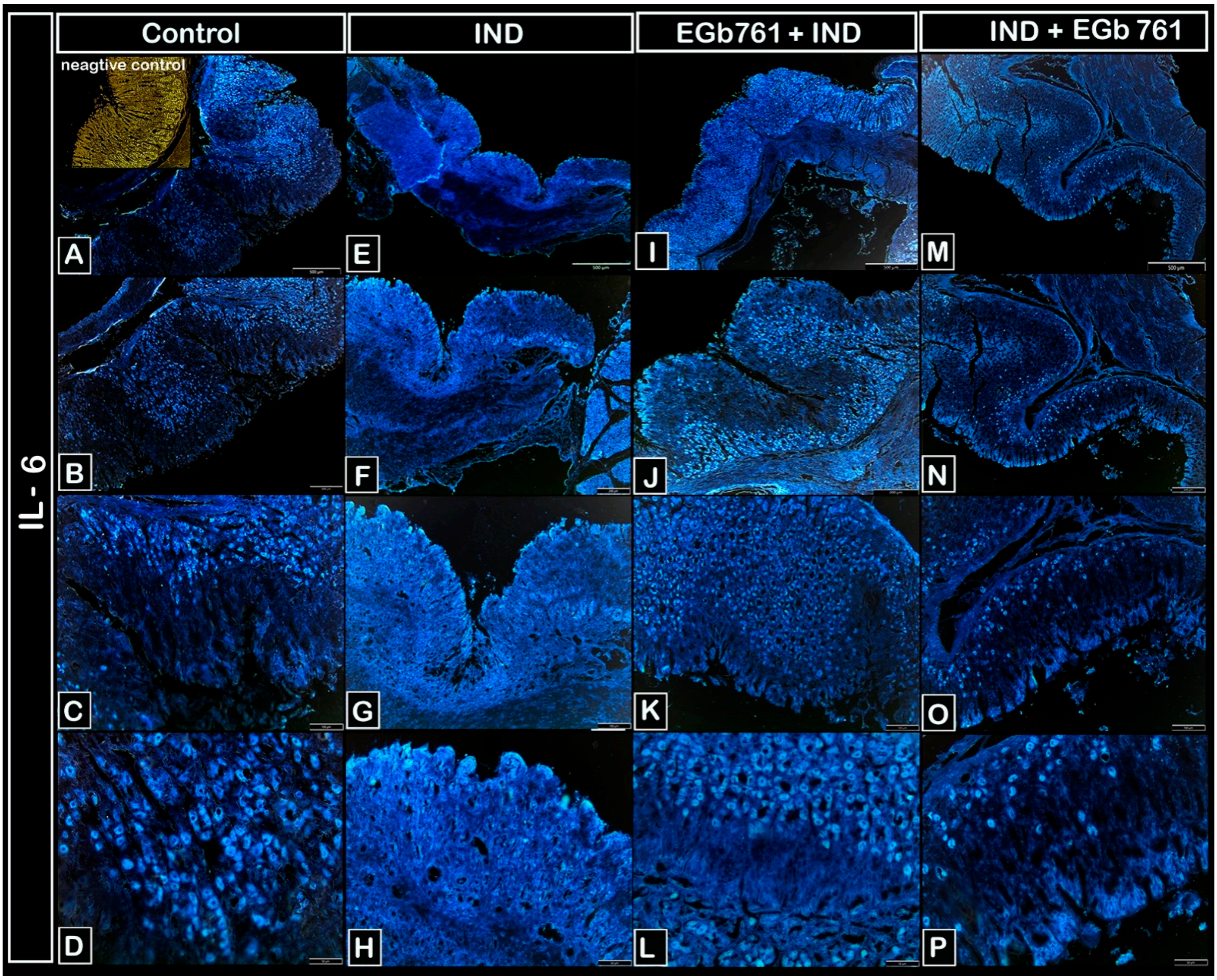

Figure S3. The negative image of the effect of indomethacin and EGb 761 on the expression of gastric IL-6. (A-D): Control mice, (E-H): Indomethacin-induced ulcer (IND), (I-L): EGb 761 pre-treatment (EGb 761+IND), (M-P): EGb 761 treatment after indomethacin (IND+EGb 761). Negative marker control is shown in the inset of figure M. IND: Indomethacin, EGb 761: Standardized Gingko biloba extract, IL-6: Interleukin-6.

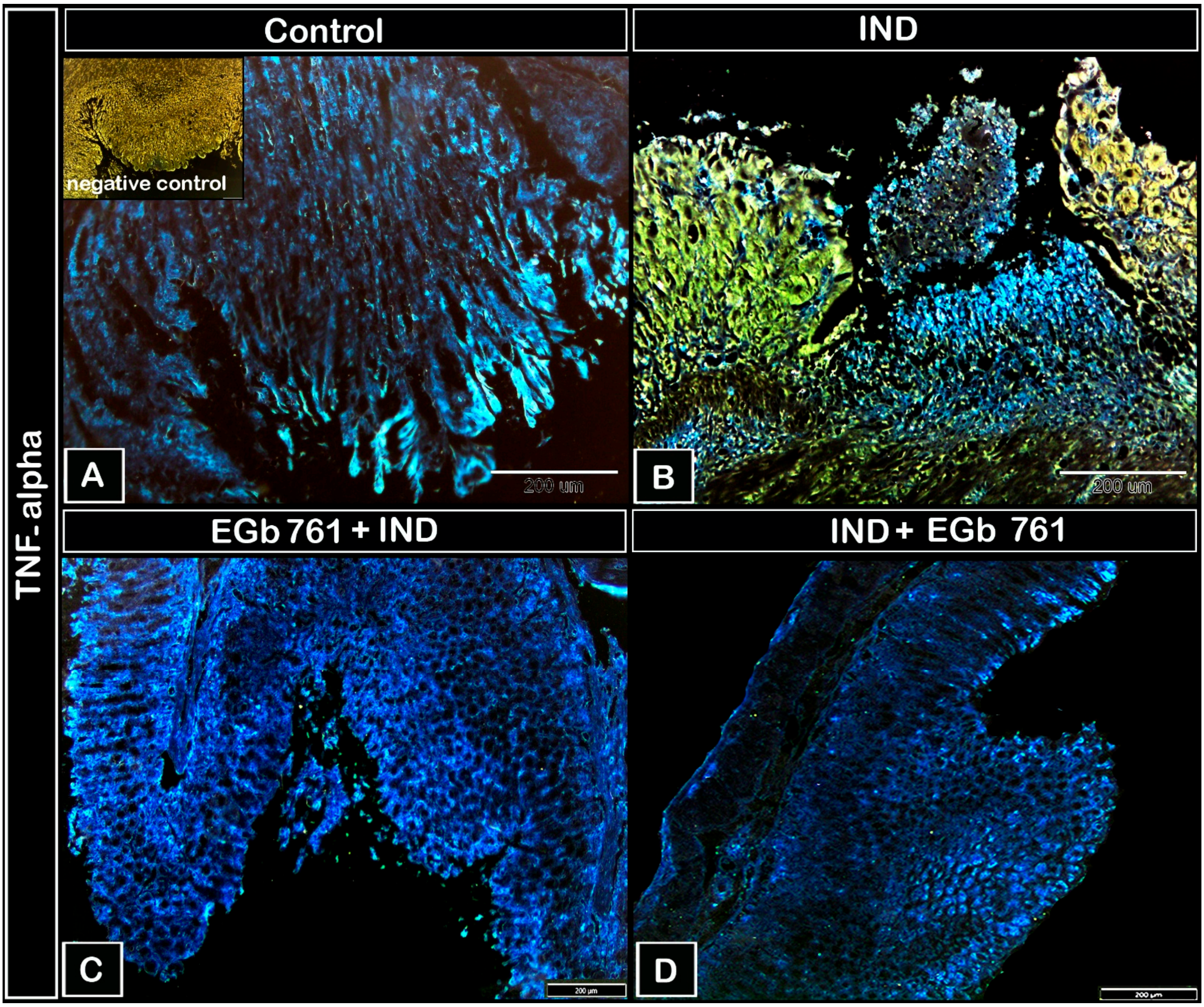

Figure S4. The negative image of changes in the gastric expression of TNF-α during indomethacin and EGb 761 administration. (A): Control mice. (B): Indomethacin-induced ulcer (IND). (C): EGb 761 pre-treatment (EGb 761+IND). (M-P): EGb 761 treatment after indomethacin (IND+EGb 761). Negative marker control is shown in the inset of figure A. IND: Indomethacin, EGb 761: Standardized Gingko biloba extract, TNF-α: Tumor necrosis factor-alpha.

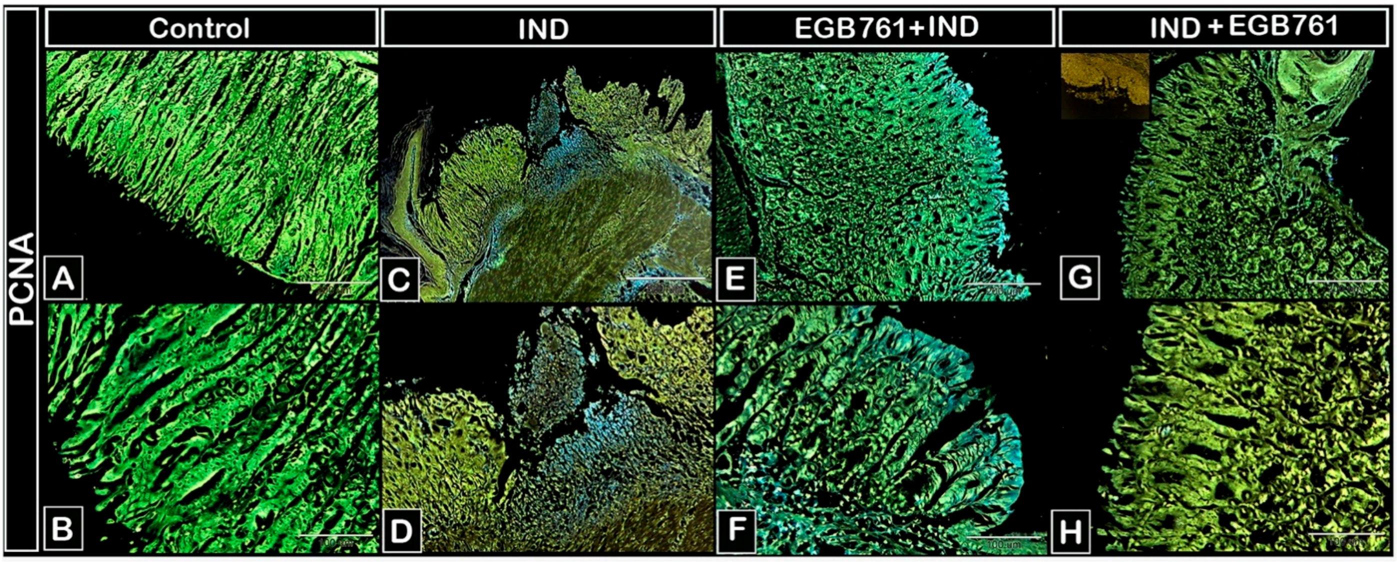

Figure S5. Negative image of the effect of indomethacin and EGb 761 on the expression of PCNA in the gastric mucosa. (A-B): Control mice. (C-D): Indomethacin-induced ulcer. (E-F): EGb 761 pre-treatment (EGb 761+IND). (G-H): EGb 761 administration after indomethacin (IND+EGb 761). Negative marker control is shown in the inset of figure G. IND: Indomethacin, EGb 761: Standardized Gingko biloba extract, PCNA: Proliferating Cell Nuclear Antigen.
